# Obesity‐induced oxidative stress and mitochondrial dysfunction negatively affect sperm quality

**DOI:** 10.1002/2211-5463.13589

**Published:** 2023-03-14

**Authors:** Jia Jing, Yuanhong Peng, Weimin Fan, Siyang Han, Qihua Peng, Chunran Xue, Xinran Qin, Yue Liu, Zhide Ding

**Affiliations:** ^1^ Department of Histology, Embryology, Genetics and Developmental Biology, Shanghai Key Laboratory for Reproductive Medicine Shanghai Jiao Tong University School of Medicine China; ^2^ Reproductive Medical Center of Ruijin Hospital Shanghai Jiao Tong University School of Medicine China; ^3^ Department of Clinical Medicine Shanghai Jiao Tong University School of Medicine China

**Keywords:** high‐fat diet (HFD), mitochondria, obesity, oxidative stress, sperm

## Abstract

Obesity is a systemic metabolic disease that can induce male infertility or subfertility through oxidative stress. The aim of this study was to determine how obesity impairs sperm mitochondrial structural integrity and function, and reduces sperm quality in both overweight/obese men and mice on a high‐fat diet (HFD). Mice fed the HFD demonstrated higher body weight and increased abdominal fat content than those fed the control diet. Such effects accompanied the decline in antioxidant enzymes, such as glutathione peroxidase (GPX) and catalase and superoxide dismutase (SOD) in testicular and epidydimal tissues. Moreover, malondialdehyde (MDA) content significantly increased in sera. Mature sperm in HFD mice demonstrated higher oxidative stress, including increased mitochondrial reactive oxygen species (ROS) levels and decreased protein expression of GPX1, which may impair mitochondrial structural integrity and reduce mitochondrial membrane potential (MMP) and ATP production. Moreover, cyclic AMPK phosphorylation status increased, whereas sperm motility declined in the HFD mice. Clinical studies demonstrated that being overweight/obese reduced SOD enzyme activity in the seminal plasma and increased ROS in sperm, accompanied by lower MMP and low‐quality sperm. Furthermore, ATP content in the sperm was negatively correlated with increases in the BMI of all clinical subjects. In conclusion, our results suggest that excessive fat intake had similar disruptive effects on sperm mitochondrial structure and function, as well as oxidative stress levels in humans and mice, which in turn induced lower sperm motility. This agreement strengthens the notion that fat‐induced increases in ROS and impaired mitochondrial function contribute to male subfertility.

## Graphical abstract

A high‐fat diet‐induced obese mouse model and clinical samples were employed to characterize the role of oxidative stress and mitochondrial dysfunction in defective spermatozoa function. Elevated reactive oxygen species (ROS) damaged mitochondrial structure and decreased the membrane potential, which resulted in detrimental feedback. Decreased ATP production and increased AMPK phosphorylation could contribute to declined sperm quality and ultimately male subfertility.

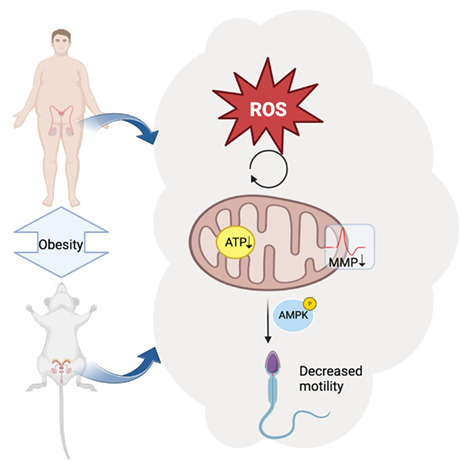

**Abbreviations**
Abasic sites (AP sites): apurinic/apyrimidinic sites
ALT: alanine aminotransferase
AMPK: AMP‐activated protein kinase
AST: aspartate transferase
BMI: body mass index
CASA: computer‐assisted sperm analysis system
CAT: catalase
CD: control diet
CHOL: cholesterol
DCFH‐DA: 2′,7′‐dihydrochlorofluorescein diacetate
DEPs: differentially expressed proteins
GPX: glutathione peroxidase
HDL‐C: high‐density lipoprotein‐cholesterol
HFD: high‐fat diet
HOMA‐IR: homeostatic model assessment for insulin resistance
JC‐1: tetrechloro‐tetraethylbenzimidazol carbocyanine iodide
LDL‐C: low‐density lipoprotein‐cholesterol
MDA: malondialdehyde
MMP: mitochondrial membrane potential
ROS: reactive oxygen species
SOD: superoxide dismutase
TEM: transmission electron microscope
TG: triglycerides
TMRM: tetramethyl rhodamine methyl ester
WST‐1: water‐soluble tetrazolium salt

Recently, ~ 39% of adults worldwide are classified as overweight and 13% are obese [1]. This pattern can impose a large healthcare economic burden on the limited available resources to adequately treat these individuals. Obesity is a systemic metabolic disease caused by genetic and environmental factors, which is associated with many diseases such as cardiovascular disease, sleep apnea, osteoarthritis, and varied types of cancer [2]. In addition, obesity can also suppress male reproductive competence due to erectile dysfunction, low testosterone levels, and infertility [3].

On the contrary, infertility has increased to astonishingly high levels worldwide and it affects about 15% of the couples seeking remediation of this condition. Among this population, males account for about 50% of the cases [4]. The association between obesity and male reproductive dysfunction has been widely reported in epidemiological studies [5, 6]. For instance, a computer‐assisted sperm analysis system (CASA) was used to analyze the semen from 1285 men. The sperm concentration and motility were all decreased, and the rates of sperm head defects and deformities were higher in obese men compared with men having normal weight [7]. Moreover, male obesity is usually associated with increased conception time and decreased likelihood of impregnation leading to pregnancy [8]. Therefore, these studies demonstrated that male obesity is closely related to decline in sperm quality.

Reactive oxygen species (ROS)‐induced sperm damage is one of the main contributing factors in 30–80% of male infertility cases [9]. Oxidative stress is defined as an imbalance between ROS production and antioxidant capacity [10]. Antioxidant systems mainly include enzymatic oxidants, such as superoxide dismutase (SOD), catalase (CAT), and glutathione peroxidase (GPX), and nonenzymatic molecules include vitamins C, pyruvate, flavonoids, carotenoids, and glutathione, etc. [6]. Sperm cells are more vulnerable to ROS due to their high polyunsaturated fatty acid content and lack of very abundant cytoplasmic antioxidases. Polyunsaturated fatty acids on the plasma membrane are the direct target of ROS. Lipid peroxidation damage of these substrates by ROS generates genotoxic end products such as malondialdehyde (MDA), and they further suppress male fertility [10]. In addition, ROS can induce sperm DNA damage, such as DNA strand breaks, introduction of apurinic/apyrimidinic (AP, abasic) sites, fragmentation and modification, etc. Both animal experiments and human studies confirmed that obesity can enhance sperm oxidative stress and DNA damage, thereby reducing its ability to induce fertilization [11, 12].

Mammalian sperm is one kind of differentiated terminal cell that has a specialized mitochondrial sheath around the middle piece of the flagellum. It serves as a source of metabolic energy to promote sperm motility. ROS generation can cause inner membrane damage of sperm mitochondria, directly impair mtDNA synthesis and disrupt the mitochondrial membrane potential (MMP) [13]. Such an effect increases the leakage of charged species from the electron transport chain leading to increased production of ROS [14]. Besides, the magnitude of the MMP is an indicator of sperm motility, which is pertinently relevant to male fertility [15]. Therefore, the extent of disruption of sperm mitochondrial function by ROS may be one of the important pathogenic factors inhibiting sperm motility.

Rats fed a high‐fat diet (HFD) in addition undergo other oxidative stress‐induced pathological changes besides decreased mitochondrial respiration efficiency in sperm, which in turn affect sperm concentration and motility [16, 17]. Ghosh and Mukherjee [18] confirmed that long‐term HFD feeding can disrupt the testicular structure, increase intracellular ROS, and enhance testicular germ cell apoptosis involving the mitochondrial intrinsic pathway. However, the mechanisms are still not fully understood that account for how obesity disrupts sperm mitochondrial structure and function and subsequently impairs the quality and motility of sperm. Herein, we show higher ROS levels and impaired mitochondrial function in mice fed with HFD. These findings have physiological relevance since they correspond to the symptomology that develops in human semen samples obtained from individuals who are in the overweight/obese group.

## Materials and methods

### Animals

Male C57BL/6 mice at 3 weeks of age were purchased from Shanghai Laboratory Animal Center (Shanghai, China). All animals were housed in the Animal Center of Shanghai Jiao Tong University School of Medicine and maintained on a 12 h light/12 h dark cycle under standard temperature (23 ± 1 °C) and relative humidity (55 ± 5%) and had free access to diet and drinking water. After acclimatization for 1 week, the mice were then randomly divided into two groups. The control diet (CD) group was continuously fed for 10 weeks a normal diet containing 19% casein, 0.2% l‐cysteine, 29.9% corn starch, 3.3% maltodextrin, 33.2% sucrose, 4.7% cellulose, 2.4% soybean oil, 1.9% lard, 4.3% mineral mix, 0.9% vitamin mix, and 0.2% choline bitartrate, with a caloric value of 3.85 kcal·gm^−1^. The other group for the same period was fed 45% high‐fat diet (HFD; Medicience, Jiangsu, China) containing 23.3% casein, 0.3% l‐cysteine, 8.5% corn starch, 11.7% maltodextrin, 20.1% sucrose, 5.8% cellulose, 2.9% soybean oil, 20.7% lard, 5.2% mineral mix, 1.2% vitamin mix, and 0.3% choline bitartrate, with a caloric value of 5.24 kcal·gm^−1^. During this period, their body weights were recorded weekly. The nose‐rump length was measured, and BMI was calculated as the body weight (g)/[nose‐rump length (cm)]^2^ [19]. The mice at 14 weeks of age were sacrificed, and then, blood, testes, caput epididymis, and cauda epididymis were collected for later analysis. Meanwhile, the liver tissue, mesenteric adipose tissue, inguinal adipose tissue, retroperitoneal adipose tissue, and epididymal adipose tissue were excised and weighted. All animal experiments and care were carefully conducted in accordance with the International Guiding Principles for Biomedical Research Involving Animals and the research program was approved by the Ethics Committee of Shanghai Jiao Tong University School of Medicine.

### Biochemical analyses

The serum was isolated from the blood by centrifuging for 15 min at 1000 × ***g***. Blood glucose concentrations were measured with a blood glucose meter (ACCU‐CHEK, Roche, Basel, Switzerland), and serum insulin was detected by an insulin ELISA kit (CRYSTAL CHEM INC, Shanghai, China). The homeostasis model assessment of insulin resistance (HOMA‐IR) index was calculated according to the formula: fasting blood glucose (mmol·L^−1^) × fasting plasma insulin (mU·L^−1^)/22.5. Meanwhile, serum lipids and liver index (total protein; albumin; ALT, alanine aminotransferase; AST, aspartate aminotransferase) were detected by Fully Automatic Biochemistry Analyzer (HITACHI 3100, Tokyo, Japan).

### Oil red O staining

Five‐micrometer thick sections were cut from frozen tissue samples with a CM1950 Cryostat (Leica, Wetzlar, Germany) and then mounted onto microscopic adhesion slides. Frozen sections were fixed with 4% PFA and then rinsed with isopropanol followed by 15 min incubation with Oil Red O solution. After incubation, sections were rinsed and stained with hematoxylin for 10 s. Finally, samples were covered with glycerine jelly and viewed under microscope (Nikon E100, Tokyo, Japan).

### Quantitative real time‐PCR (RT‐qPCR) analysis

Total RNA was extracted from testes, caput epididymis, and cauda epididymis. The cDNA was synthesized using Reverse Transcription kit (Takara, Tokyo, Japan) according to the manufacturer's protocol. RT‐qPCR was performed on Applied Biosystem 7500 using the SYBR Green PCR kit (Takara) and the cycling conditions included 30 s incubation at 95 °C, followed by 40 cycles at 94 °C for 5 s and 60 °C for 30 s. Data were normalized to β‐actin, respectively. Sequences of primers used for RT‐qPCR were as follows: *Actin*, 5′‐GTGACGTTGACATCCGTAAAGA‐3′ and 5′‐GCCGGACTCATCGTACTC‐3′; *Sod1*, 5′‐CAGCATGGGTTCCACGTCCA‐3′ and 5′‐CACATTGGCCACACCGTCCT‐3′; *Gpx*, 5′‐GGGCAAGGTGCTGCTCATTG‐3′ and 5′‐AGAGCGGGTGAGCCTTCTCA‐3′; *Cat*, 5′‐CCAGCGACCAGATGAAGCAG‐3′ and 5′‐CCACTCTCTCAGGAATCCGC‐3′.

### Sperm parameters measurement in mice

The caudal region of epididymis in each mouse was dissected out without fat tissue and cut into pieces in prewarmed (37 °C) Tyrode's buffer (Sigma‐Aldrich, St. Louis, MO, USA). Sperm were allowed to diffuse into the medium for 15 min at 37 °C, and then, sperm suspension was collected in a new Eppendorf tube. Finally, the sperm concentration, motility, and progressive motility were analyzed by a computer‐assisted sperm analysis system (CASA; Hamilton Thorne, Beverly, MA, USA).

### Colorimetric assay measurement of the SOD activity

Mouse testes, caput epididymis, and cauda epididymis were collected and washed with saline to remove blood. Then, 500 μL of a sucrose buffer (0.25 mm sucrose, 10 mm HEPES, 1 mm EDTA, pH 7.4) were added to the above‐mentioned tissues that were then homogenized. After centrifugation (10 000 × ***g***, 60 min, 4 °C), the supernatants were immediately transferred into new tubes. Clinical semen samples were centrifuged at 800 × ***g*** for 5 min and the upper seminal plasma was also transferred into new tubes. Cu/Zn SOD inhibitors supplied in the kit were added to the samples used to measure Mn‐SOD activity.

A SOD Assay kit (Dojindo, Tabaru, Japan) was used to measure SOD activity by utilizing a highly water‐soluble tetrazolium salt (WST‐1), which produces a water‐soluble formazan dye upon reduction with the superoxide anion. Briefly, a 20 μL sample aliquot was added to each well on a plate and a WST‐1 containing working solution was added, and then mixed with enzyme working solution. Afterward, the plate was incubated at 37 °C for 20 min and the optical absorbance of each well was read at 450 nm. SOD activity (inhibition rate %) was calculated using the provided equation in the protocols.

### MDA assay in mouse serum

Blood samples were taken from the mouse heart under anesthesia and left at room temperature for 30 min, followed by centrifugation at 1500 × ***g*** for 15 min at 4 °C. Serum was transferred to a new polypropylene tube. One hundred and fifty microliter serum samples were added to centrifuge tubes and mixed with the reaction buffer described in the MDA Assay kit (Jian Cheng Bioengineering Institute, Nanjing, China), and then incubated at 95 °C for 60 min. After centrifuging for 10 min at 3200 × ***g***, a colorimetric assay was used to immediately measure and evaluate with a microplate reader the optical absorbance at 532 nm. The values of the blank were subtracted from all samples and standard readings. A standard curve was used to calculate the MDA concentration in each sample.

### DNA damage quantification

Mouse sperm were isolated by centrifugation in a 40% Percoll gradient, and DNA was extracted with a DNA extraction kit (Omega Biotek, Norcross, GA, USA). The abasic sites were quantified using the DNA damage quantification‐AP site counting kit (Dojindo). Briefly, a mix containing 10 μL of purified genomic DNA solution (100 μg·mL^−1^) and 10 μL of ARP solution in a 0.5 mL tube was incubated at 37 °C for 1 h, which was followed by purification of the ARP‐labeled DNA. Diluted ARP‐labeled DNA and standards were mixed with DNA binding solution, and then bound with HRP‐Streptavidin, and the O.D. was measured at 650 nm. Determination of the number of abasic sites in the DNA used the calibration curve.

### ATP levels measurement in sperm

ATP levels in the sperm were measured using an ATP assay kit (Abcam, Boston, MA, USA) according to the manufacturer's instructions. Briefly, 3 × 10^7^ sperm were collected and washed with cold phosphate buffer saline (PBS) thrice, then the sperm suspension was centrifuged (500 × ***g***, 5 min) and the supernatant was removed. The pellet was resuspended in ATP assay buffer to extract ATP, and then, samples were centrifuged at 13 000 × ***g*** for 5 min at 4 °C to remove any insoluble material. The supernatant was transferred into new tubes, and a 50 μL sample was added into each well in a plate and mixed with ATP reaction buffer. Then, the plate was incubated at 37 °C for 30 min in darkness and the optical absorbance of each well was read at 570 nm. The mean value of the blank was subtracted from all standards and sample readings. A standard curve was used to calculate the ATP concentration in each sample.

### Proteomics analysis

Mouse sperm were isolated by centrifugation in a 40% Percoll gradient and then prepared for liquid chromatography–tandem mass spectrometry (LC–MS). Proteomics analysis was performed as previously described [20]. Differentially expressed proteins (DEPs) associated with mitochondrial function were identified using mouse mitocarta 3.0 (https://www.broadinstitute.org/mitocarta).

### Transmission electron microscope (TEM) analysis

To observe the ultra‐structure of sperm mitochondria, small pieces from the cauda epididymis of mice were fixed in 2% glutaraldehyde for 2 h at 4 °C. Then, the tissues were postfixed with 1% OsO4 for 2 h at 4 °C, and later dehydrated through sequential washes with an ascending series of ethanol concentrations and then embedded in Araldite. Ultrathin sections were stained with uranyl acetate and lead citrate and analyzed by transmission electron microscopy (Hitachi, Tokyo, Japan).

### Fluorescent staining and flow cytometry

Mouse sperm (2 × 10^6^ sperm) were collected from the cauda epididymis and washed with PBS thrice, and then incubated with a fluorescence probe in darkness. Liperfluo (Dojindo), indicator of lipid peroxidation, was added to each sample at a final concentration of 2 μm and incubated for 30 min at 37 °C. A Si‐DMA kit (Dojindo) evaluated mitochondrial singlet oxygen (^1^O_2_) levels in samples that were incubated at a final concentration of 50 nm for 45 min at 37 °C. For sperm analysis of the cellular ROS and mitochondrial membrane potential (MMP), DCFH‐DA (Yeasen, Shanghai, China; magnification = 1000×), JC‐1 (Yeasen; magnification = 200×), and TMRM (M20036, Invitrogen, Waltham, MA, USA; magnification = 1000×) probes were incubated at 37 °C for 30 min. After incubation, samples were washed twice and resuspended in PBS for flow cytometry assay (Cytoflex S flow cytometer, Beckman, CA, USA). A total of 10 000 events were collected for measuring fluorescence intensity.

Meanwhile, after fluorescent staining, 20 μL of a sperm suspension was smeared on a clean slide glass and covered with 22 mm square‐shaped coverslips. Fluorescent staining of Liperfluo (Ex: 488 nm) and si‐DMA (Ex: 640 nm) in sperm were also recorded under an upright fluorescent microscope (Zeiss 710, Jena, Germany).

### Western blot analysis

Sperm were collected from mouse caudal epididymis by centrifugation in a 45% Percoll gradient at 800 × ***g*** for 20 min at 4 °C and then washed thrice with ice‐cold PBS. They were then lysed in lysis buffer (6 m urea, 2 m thiourea, and 4% CHAPS). After centrifugation (12 000 × ***g***, 20 min) at 4 °C, the supernatants were collected and concentrated. SDS/PAGE was performed, and then, the sperm proteins were electroblotted onto polyvinylidene difluoride (PVDF) membranes (Millipore, Burlington, MA, USA). The membranes were blocked with TBST (10 mm Tris‐HCl, pH 8.0, 150 mm NaCl, 0.1% Tween‐20) containing 5% bovine serum albumin (BSA) for 1 h at room temperature, followed by incubation with the primary antibodies, such as anti‐GPX1, anti‐AMPK, anti‐p‐AMPK, anti‐β‐actin, anti‐Tubulin (Cell Signaling Technology, Danvers, MA, USA) overnight at 4 °C. After washing with TBST thrice, membranes were incubated with horseradish peroxidase (HRP)‐conjugated anti‐mouse or anti‐rabbit antibody (Abgent, San Diego, CA, USA) for 1 h at room temperature. Then, enhanced chemiluminescence was employed to generate the signals, which were detected by luminescent image analyzer (Image Quant LAS 4000, Chicago, IL, USA). The densitometric quantification of each protein band was performed using imagej software (National Institutes of Health, NIH, Bethesda, MD, USA).

### Human semen samples preparation and analyses

All human semen samples and relevant clinical data were collected from the Reproductive Medicine Center, Ruijin Hospital, Shanghai Jiao Tong University School of Medicine. This study conformed to the standards set by the Declaration of Helsinki and was approved by the Institutional Review Boards of Shanghai Ruijin Hospital, and all subjects (20–35 years old) participating in the study provided informed written consent for the use of their leftover semen samples when all IVF treatments finished. According to the WHO classification criteria, subjects were divided into the normal weight group (*n* = 46, 18.5 ≤ BMI < 25 kg·m^−2^) and the overweight/obese group (*n* = 69, BMI ≥ 25 kg·m^−2^). Notably, individuals having a history of long‐term medication, varicocele, and infection as indicated by a large number of leukocytes in the semen were excluded from the study. Furthermore, samples that were hyperviscous and necrozoospermic (sperm viability < 70%) were also excluded from the study.

The semen samples were placed at 37 °C for liquefaction. The volume and pH value of semen were measured, and sperm parameters were analyzed by CASA (Hamilton‐Thorn Research, Beverly, MA, USA) and then evaluated based on parameters described in the World Health Organization guidelines (WHO, 5^th^, 2010).

Fresh human semen samples were centrifuged at 800 × ***g*** for 20 min, and subsequently, seminal plasma was used for the analysis of SOD activity and the sperm precipitates were washed twice with PBS for ATP measurement by flow cytometry analysis according to described methods.

### Statistical analyses

All experimental data are expressed as numbers of replicates and mean ± standard deviation (SD). The statistical analyses were conducted with IBM spss Statistics 25.0 (IBM Corp, Armonk, NY, USA). Comparison of continuous data between two groups was made using the unpaired Student's *t*‐test appropriately. Levene's statistics were used to test for homogeneity of variance. A normal probability plot was used to check for the distribution of the data. Comparison of body weight between two groups at different time points was done using a two‐way analysis of variance (ANOVA) with the Bonferroni–Dunn's multiple comparison test. The relationship between two sets of data in the human study was evaluated using the Pearson correlation coefficient and simple linear regression method. *P* < 0.05 was considered statistically significant.

## Results

### Increases in body weight and abdominal adipose tissue in HFD mice

**Fig. 1:**
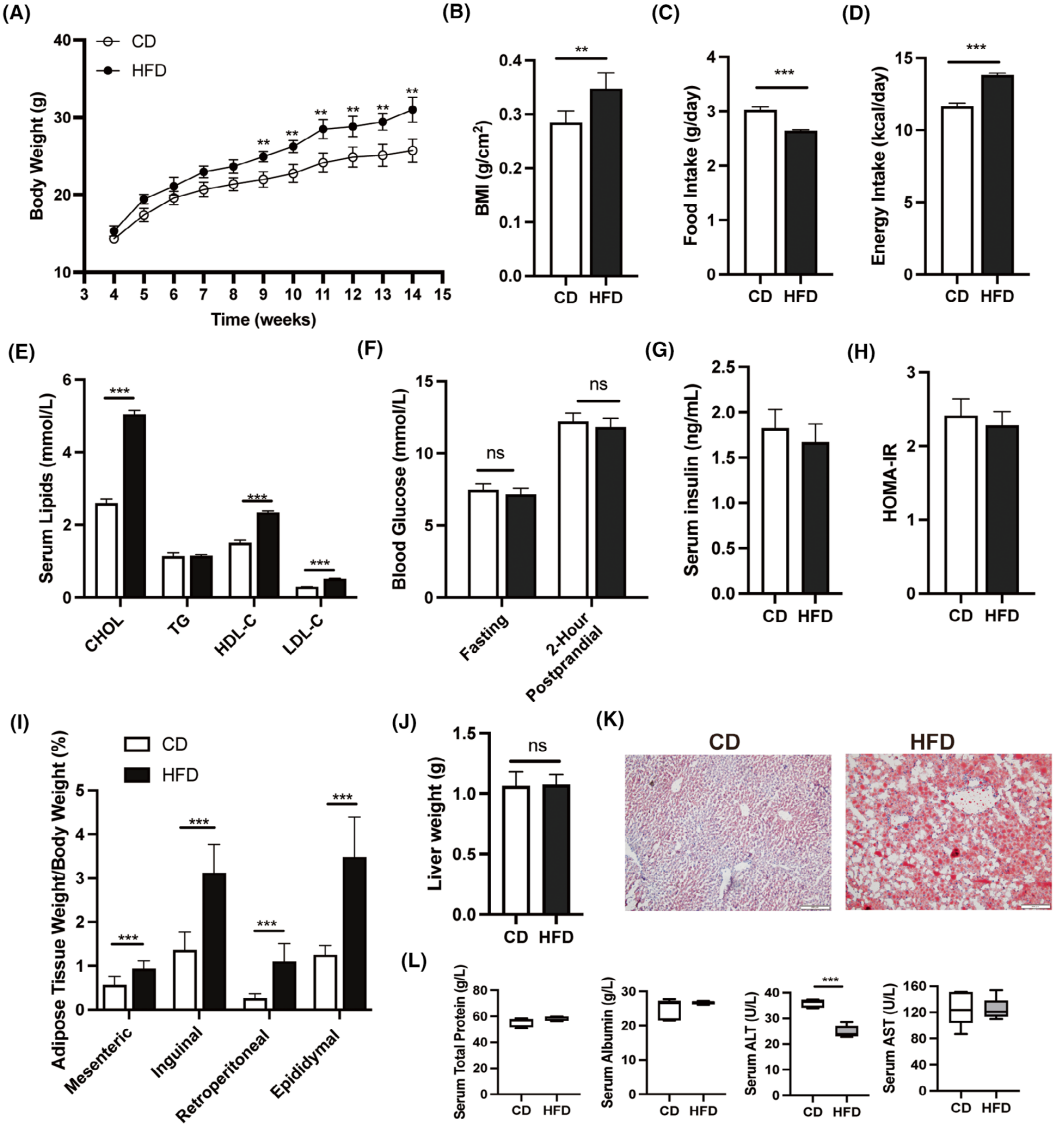
Effects of high‐fat diet on body weight, daily food intake, liver weight, and different abdominal adipose tissues in mice. (A) Comparison of the body weight between the control diet (CD) and high‐fat diet (HFD) mice from 4 to 14 weeks of age (*n* = 20). (B–D) Comparison of BMI, daily food intake, and energy intake in CD and HFD mice at 14 weeks of age. (E) Serum lipids detection. (F–H) Fasting blood glucose, 2‐h postprandial blood glucose levels, serum insulin levels, and HOMA‐IR in mice. (I) The ratio of the weight of mesenteric, inguinal, retroperitoneal, and epididymal adipose tissue to body weight, respectively, in CD and HFD mice at 14 weeks of age (*n* = 6). (J) Liver weight. (K) Oil Red O staining of liver tissues. Scale bars, 200 μm. (L) The levels of total protein, albumin, ALT, and AST in mouse serum (*n* = 6). Data are expressed as mean ± SD. Statistical analyses were performed using the two‐way ANOVA (A) and Student's *t*‐test (B–J, L) NS, no significant difference. **P < 0.01, ***P < 0.001. ALT, alanine aminotransferase; AST, aspartate transferase; BMI, body mass index; CHOL, cholesterol; HDL‐C, high‐density lipoprotein cholesterol; HOMA‐IR, homeostatic model assessment for insulin resistance; LDL‐C, low‐density lipoprotein cholesterol; TG, triglycerides.

Male C57BL/6 mice were continuously fed with 45% high‐fat diet from 4^th^ week for 10 weeks to establish the diet‐induced obese mouse model. Their weight gain was more than that in the control diet (CD) group from the 9 to 14 weeks of age (Fig. 1A). Notably, the BMI of HFD mice was significantly higher than that in matched CD group (0.35 ± 0.03 vs. 0.28 ± 0.22 g, *P* < 0.01) at 14th week (Fig. 1B). Daily food intake in HFD mice was lower than that in CD mice (Fig. 1C). However, the daily energy intake increased in HFD group (Fig. 1D). Serum lipids also showed higher levels of cholesterol, HDL‐C, and LDL‐C (Fig. 1E) as reported in previous data [21]. Besides, the HFD had no effect on fasting and postprandial blood glucose levels in mice (Fig. 1F). Meanwhile, there were no significant differences in the serum insulin levels and HOMA‐IR between the two groups (Fig. 1G,H). Compared with the CD group, HFD mice revealed enlarged mesenteric adipose tissue (0.94 ± 0.18 vs. 0.57 ± 0.19%, *P* < 0.01), inguinal adipose tissue (3.12 ± 0.65 vs. 1.36 ± 0.41%, *P* < 0.001), retroperitoneal adipose tissue (1.1 ± 0.41 vs. 0.26 ± 0.11%, *P* < 0.001), and epididymal adipose tissue (3.48 ± 0.91 vs. 1.25 ± 0.22%, *P* < 0.001; Fig. 1I). Even though the liver weight showed no change in the current study, fatty liver, and lower serum ALT levels were observed in the HFD mice (Fig. 1J–L). Therefore, these results undoubtedly indicate that adipose tissues gain appreciable amounts of fat in multiple organs including the male reproductive organs in the HFD group.

### Decreased expression of antioxidant enzymes in reproductive tissues

**Fig. 2:**
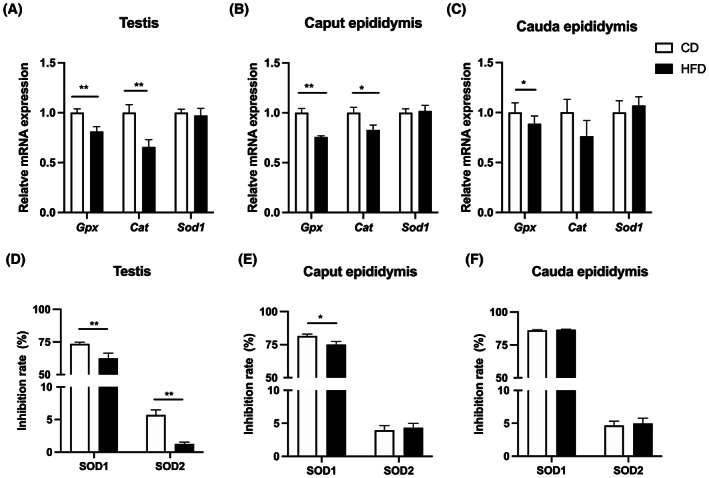
Expression of antioxidative enzymes in mouse reproductive tissues. The relative mRNA expression levels of antioxidants (GPX, Catalase and SOD1) in testis (A), caput of epididymis (B), and cauda of epididymis (C) from CD and HFD mice. (D–F) Comparisons of SOD1 and SOD2 enzyme activities in testis, caput of epididymis, and cauda of epididymis between CD and HFD mice, shown as percentage (%). Data are expressed as mean ± SD (*n* = 10). Statistical analysis was performed using the Student's *t*‐test. *P < 0.05; **P < 0.01. GPX, glutathione peroxidase; SOD, superoxide dismutase.

To determine whether the HFD induces an increase in oxidative stress status, we analyzed the antioxidant gene expression levels of three representative enzymes in the reproductive tissue. They include glutathione peroxidase (GPX), catalase (CAT), and superoxide dismutase (SOD) in the testes, caput epididymis, and cauda epididymis. The q‐RT PCR results showed that GPX and CAT had lower mRNA expression levels in both the testes and caput epididymis in the HFD mice than those in the CD group (Fig. 2A,B), whereas only GPX had a lower gene expression level in the cauda epididymis in HFD mice (Fig. 2C). However, the SOD1 mRNA expression levels were not different between two groups in the reproductive tissue (Fig. 2A–C). SOD1, also known as CuZn‐SOD, is one type of SOD expressed in the cytosol, whereas SOD2 (Mn‐SOD) is another type of SOD localized in the mitochondria. Considering the discrepancy between SOD gene expression and its enzymatic activity, we found that in the HFD mice, both the SOD1 and SOD2 enzymatic activities decreased in the testes (Fig. 2D), and the SOD1 enzymatic activity also exhibited a slight decrease in the caput epididymis (Fig. 2E); however, the two enzymatic activities were invariant in the cauda epididymis of these two groups (Fig. 2F). Therefore, these results suggested that antioxidant capacity decreased in reproductive tissues of HFD mice.

### Increased ROS in HFD mice sperm

**Fig. 3:**
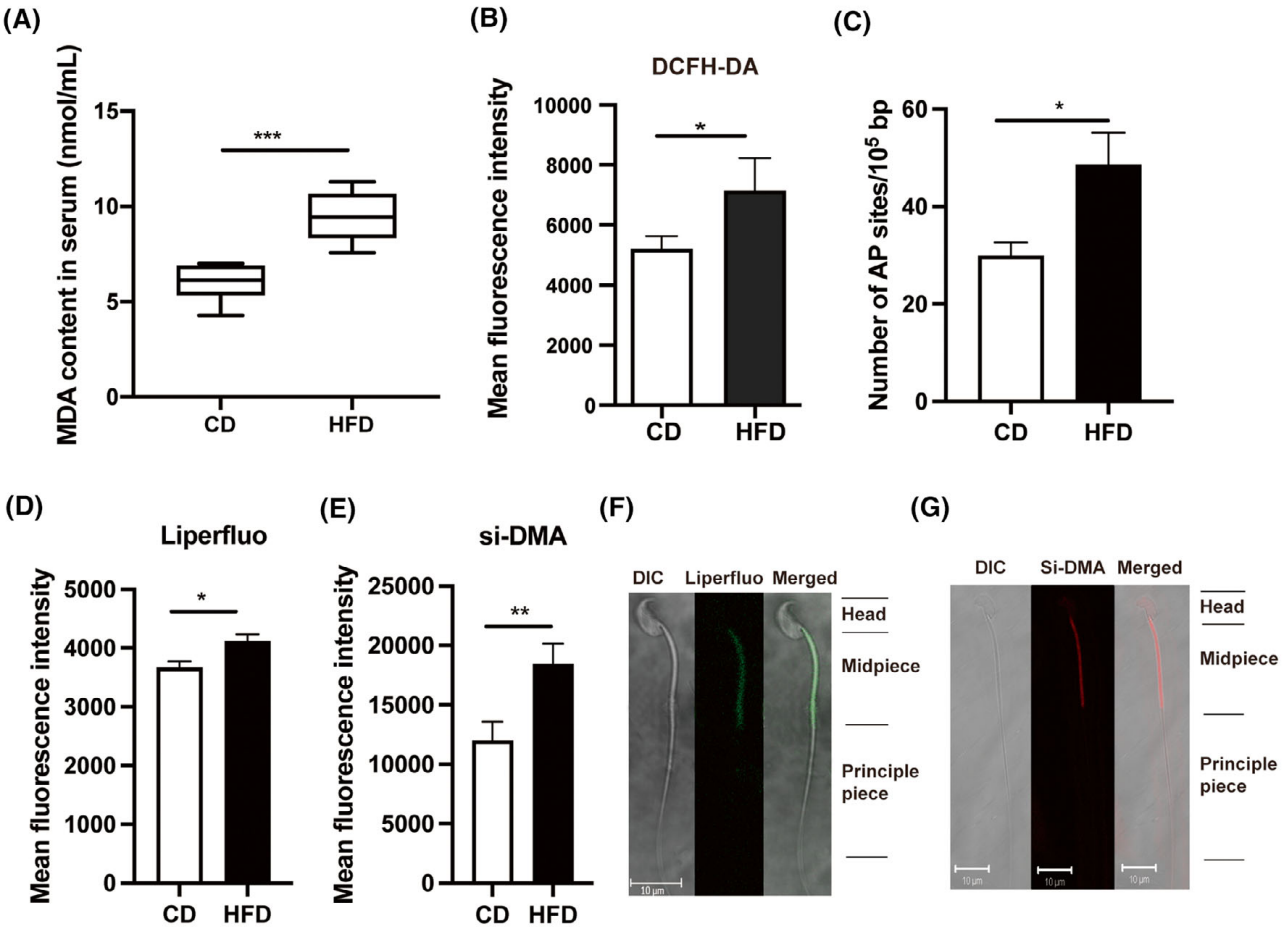
Oxidative stress‐induced changes in mouse serum and sperm. (A) The MDA content in serum from HFD mice was significantly higher than that in the CD group (*n* = 6). (B) ROS levels in mouse sperm were measured by fluorescent dye (DCFH‐DA). (C) DNA damage quantification in mouse sperm, AP sites: apurinic/apyrimidinic sites. (D, E) Comparisons of mean fluorescence intensity of Liperfluo and si‐DMA in mouse sperm between CD and HFD groups (*n* = 10). (F, G) Liperfluo and si‐DMA staining in mouse sperm were visualized by confocal fluorescence microscopy. Scale bars, 10 μm. Data are expressed as mean ± SD. Statistical analysis was performed using the Student's *t*‐test. *P < 0.05; **P < 0.01; ***P < 0.001. DCFH‐DA, 2′,7′‐dihydrochlorofluorescein diacetate; Liperfluo and si‐DMA, fluorescent probes for detecting lipid peroxidation and mitochondrial singlet oxygen, respectively; MDA, malondialdehyde.

Malondialdehyde (MDA), one of the final products of polyunsaturated fatty acids peroxidation, is an index of lipid peroxidation [22]. Compared with the CD group, the concentration of MDA in the serum of the HFD mice increased significantly (9.49 ± 1.35 vs. 6.02 ± 0.98 nmol·mL^−1^, *P* < 0.001, Fig. 3A). Thus, the HFD induced a maladaptive response that led to oxidative stress through suppressing antioxidant gene expression in both the testes and epididymis, which could disrupt spermiogenesis and sperm maturation. On the contrary, HFD‐fed mice had an increased ROS level and DNA oxidative damage in their sperm (Fig. 3B,C). To clarify in more detail how oxidative stress induces pathological changes in the mature sperm of the HFD mice, the underlying changes in the lipid peroxidation (Liperfluo) and mitochondrial single oxygen (si‐DMA) levels were evaluated with fluorescent probes. The results of fluorescent staining showed that the mean fluorescence intensity of these two molecular probes was significantly elevated in the sperm of mice on the HFD relative to that in the age‐matched CD group (Fig. 3D,E). Moreover, the confocal imaging showed that lipid peroxidation and mitochondria single oxygen levels rose in the midpiece of sperm (mitochondrial sheath; Fig. 3F,G). These results strongly indicate that HFD can induce higher ROS levels, which induce oxidative attacks on the lipids and DNA in mouse sperm.

### Impaired sperm mitochondrial structure and function in HFD mice

**Fig. 4:**
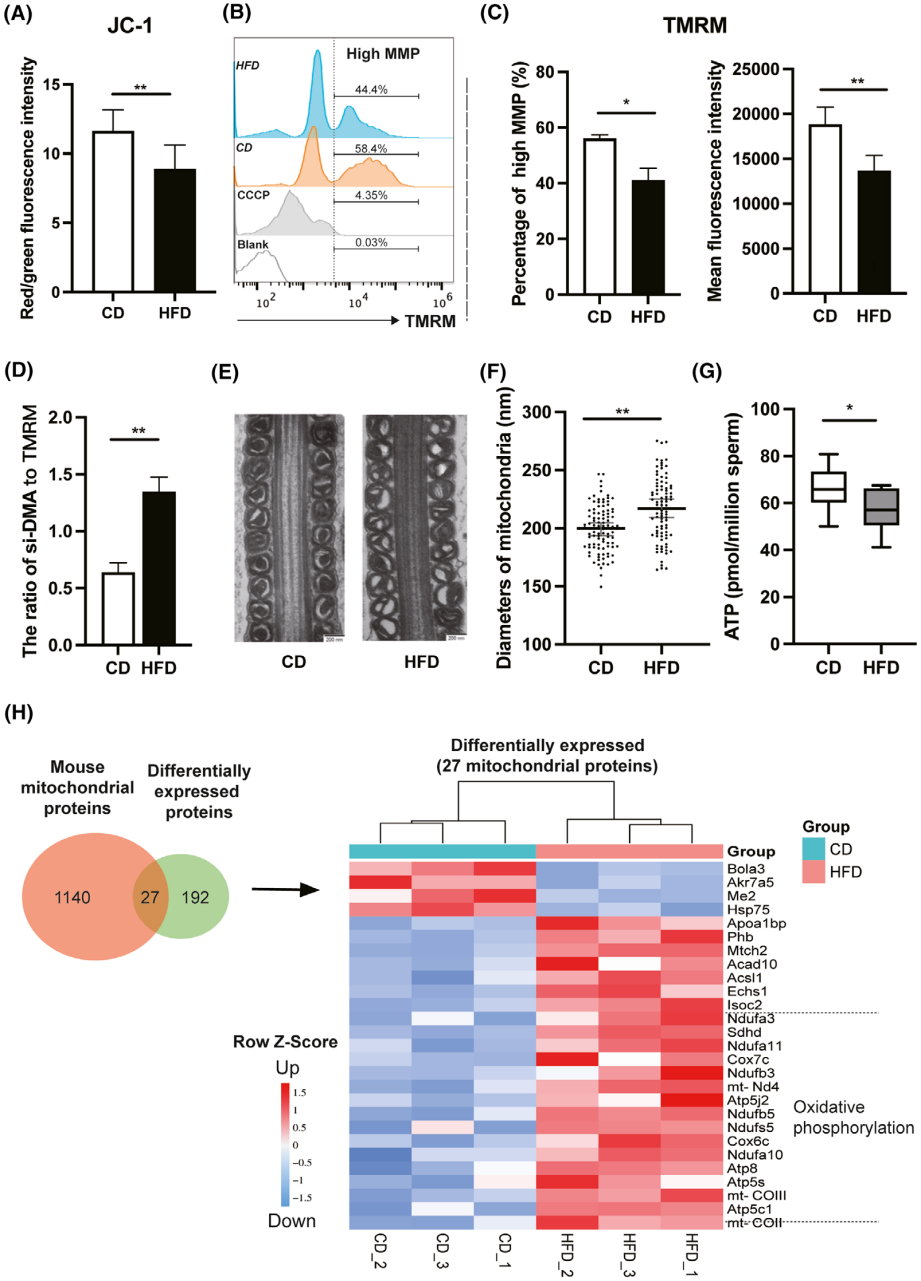
Mitochondrial structural and functional analyses of mouse sperm. Mitochondrial membrane potential (MMP) of sperm in mice based on measurement of the JC‐1 red/green fluorescence ratio (A) and TMRM staining (B and C, CCCP as a positive control). (D) The ratio of si‐DMA fluorescence intensity to TMRM fluorescence intensity. (E) Mitochondrial morphology of sperm from cauda epididymis in CD and HFD mice was observed by transmission electron microscope (TEM). Scale bars, 200 nm. (F) Statistical analysis of sperm mitochondrial diameters. (G) Comparison of sperm ATP content between CD and HFD mice. (H) Comparative proteomics analysis of differentially expressed proteins (DEPs) relevant to mitochondrial function in sperm between CD and HFD mice. Data are expressed as mean ± SD (*n* = 10). Statistical analysis was performed using the Student's *t*‐test. *P < 0.05; **P < 0.01. JC‐1, tetrechloro‐tetraethylbenzimidazol carbocyanine iodide; TMRM, tetramethyl rhodamine methyl ester.

Mitochondria conserve metabolic energy through the generation of ATP, which is needed to support sperm mobility. Higher superoxide in mitochondria may contribute to damage of the mitochondrial inner membrane and reduced MMP [23]. We determined whether the alterations in sperm integrity and quality induced by ROS generation stem from disruption of mitochondrial structure and function in the mice fed on HFD. JC‐1 and TMRM probes were used for analyzing MMP in the sperm [24]. Generally, JC‐1 forms J‐aggregates at higher MMP with red fluorescence, whereas exists as a monomer emitting green fluorescence when mitochondria exhibit low MMP. In our case, the ratio of red fluorescence to green fluorescence with JC‐1 staining, which reflects the mitochondrial membrane potential, was significantly declined in HFD mouse sperm (Fig. 4A). Meanwhile, TMRM can also detect sperm populations displaying either high or low MMP, which correlates with sperm quality [25]. The percentage of cells with high MMP decreased in HFD mice compared with that in the CD group, indicating a lower mean TMRM immunofluorescence staining intensity (Fig. 4B,C). Considering that si‐DMA probes accumulated in mitochondria are MMP‐dependent, we normalized si‐DMA fluorescence intensity to TMRM fluorescence intensity, and the results further indicated higher levels of mitochondrial single oxygen in sperm from HFD mouse (Fig. 4D). Next, we continued to evaluate changes in sperm mitochondrial structure. The results of transmission electron microscopy (TEM) showed that the mitochondrial morphology was disrupted in the mature sperm of cauda epididymis. The notable changes include swelling, vacuolation, and disordered arrangement of mitochondrial cristae in the HFD mice (Fig. 4E,F). Moreover, the sperm ATP content in the HFD mice significantly decreased compared with that in the CD group (56.72 ± 9.46 vs. 66.46 ± 9.06 pmol per 10^6^ sperm, *P* < 0.05, Fig. 4G). On the contrary, proteomics analysis of sperm identified differentially expressed proteins (DEPs) [20]. The changes relevant to the mitochondrial function showed that the proteins involved in regulating oxidative phosphorylation status were upregulated in HFD‐fed mice, which may be a compensatory mechanism in sperm to offset declines in energy transduction (Fig. 4H). Accordingly, these results show impaired mitochondrial structure and function in sperm from HFD mice.

### HFD reduces sperm quality through decreased GPX1 expression, increased AMPK phosphorylation status, and declined ATP accumulation

**Fig. 5:**
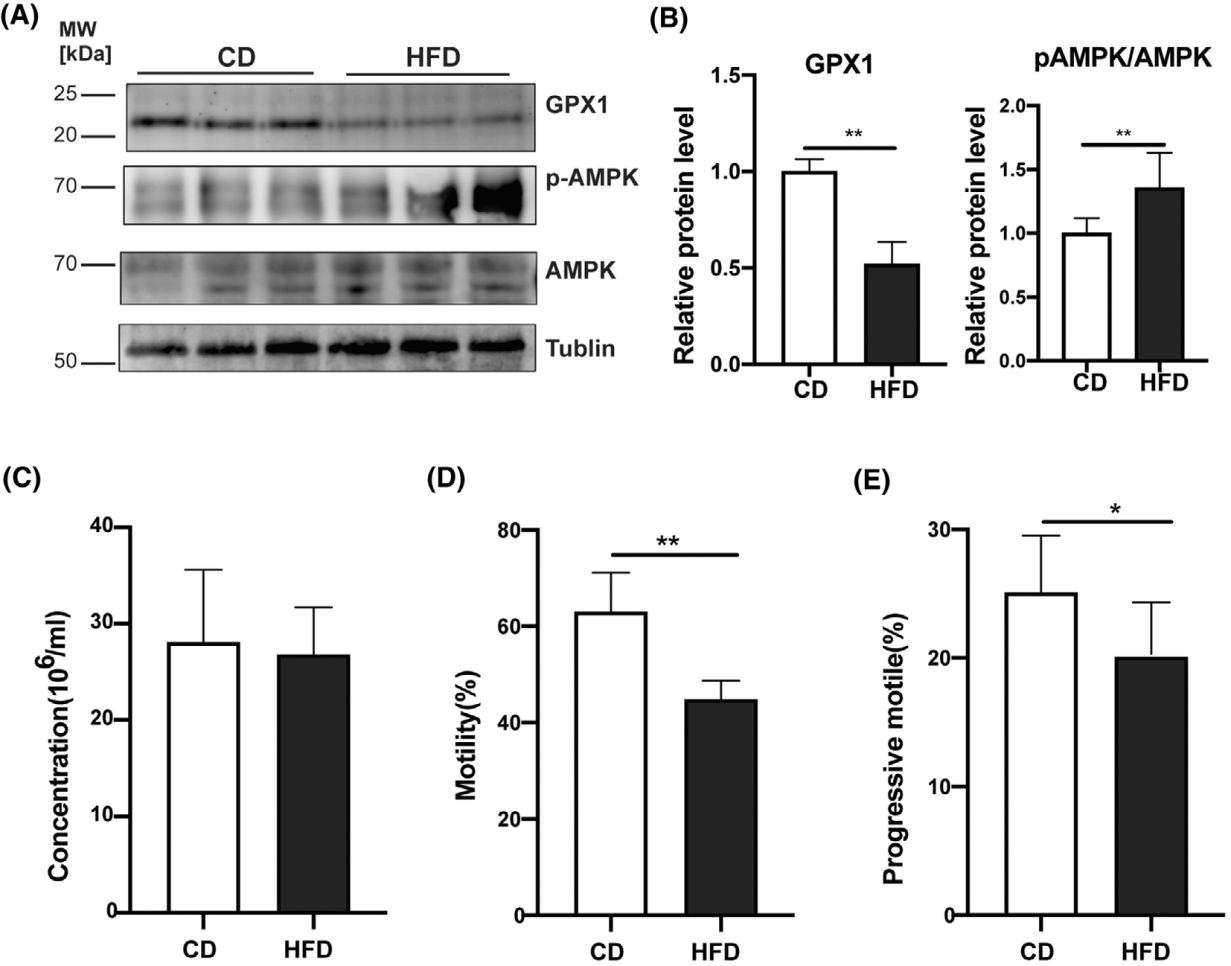
Sperm GPX1, AMPK, and p‐AMPK expressions and sperm parameters in mice. (A) Western blot analyses of protein expression levels of GPX 1, AMPK, and p‐AMPK (Thr‐172) in sperm from CD and HFD mice. Tubulin was used to validate protein loading equivalence simultaneously. (B) The quantification of protein bands in (A), shown as relative protein expression level (*n* = 8). (C–E) Comparison of sperm parameters in CD and HFD mice, including sperm concentration, motility, and progressive motility (*n* = 10). Data are expressed as mean ± SD. Statistical analysis was performed using the Student's *t*‐test. *P < 0.05; **P < 0.01. AMPK, adenosine 5′‐monophosphate‐activated protein kinase; GPX1, glutathione peroxidase 1; p‐AMPK, phosphorylation of AMP‐activated protein kinase.

The AMPK activity is a vital support for maintaining sperm motility, which can be activated by either oxidative stress or decreases in ATP generation [26, 27]. GPX1 is a glutathione peroxidase that protects cells from oxidative stress and it is widely expressed in many tissues. It detoxifies hydrogen peroxide, specifically by catalyzing its reduction to water. Western blot analysis revealed that GPX1 protein expression decreased and AMPK Threonine‐172 phosphorylation status significantly increases in mature sperm from cauda epididymis of HFD mice compared with those in the CD group (Fig. 5A,B). On the contrary, CASA was employed to evaluate the impact of such changes on sperm quality. The results showed that the motility and progressive motility of sperm isolated from the HFD group decreased significantly compared with that from the CD group, respectively (44.80 ± 3.82 vs. 63.10 ± 0.08%, *P* < 0.01; 20.10 ± 4.01 vs. 25.10 ± 4.40%, *P* < 0.05, Fig. 5D,E). By contrast, the sperm concentrations remained similar to one another in both the HFD and CD groups (26.81 ± 4.87 vs. 28.09 ± 7.49·10^6^ mL^−1^, *P* > 0.05, Fig. 5C). Accordingly, these results demonstrate that both persistent exposure to oxidative stress and the declines in ATP generation may induce rises in AMPK activity, which damage sperm quality and its motility.

### Damaged mitochondrial function induced by increased oxidative stress in clinical overweight/obese subjects

A total of 115 men were enrolled in this study, including 46 normal weight men whose BMI values were placed in a control group (22.85 ± 1.45 kg·m^−2^) whereas 69 others were assigned to the overweight/obese group (28.54 ± 3.05 kg·m^−2^; *P* < 0.001). There were no statistical differences in both male height and age between these two groups. Considering seminal parameters, the semen volume (3.67 ± 1.38 vs. 3.51 ± 1.33 mL, *P* > 0.05), sperm concentration (106.36 ± 55.08 vs. 91.14 ± 75.25·10^6^ mL^−1^, *P* > 0.05), and semen pH (7.48 ± 0.1 vs. 7.48 ± 0.09, *P* > 005) were also not different between the normal weight and overweight/obese men (Table 1).

**Table 1:** Clinical data and semen parameters in normal weight and overweight/obese subjects. BMI, Body Mass Index; values represent mean ± SD; *P* < 0.05 represents statistical significance.

Group	*n*	Height (m)	BMI (kg·m^−2^)	Age (years)	Semen volume (mL)	Sperm concentration (10^6^ mL^−1^)	Semen, pH	Sperm motility (%)
Normal	46	1.73 ± 0.07	22.36 ± 1.85	31.52 ± 4.48	3.67 ± 1.38	106.36 ± 55.08	7.48 ± 0.10	74.88 ± 12.48
Overweight/obesity	69	1.75 ± 0.06	28.41 ± 3.08	32.78 ± 4.46	3.51 ± 1.33	91.14 ± 75.25	7.48 ± 0.09	62.29 ± 22.46
*P* value		0.093	< 0.001	0.143	0.537	0.243	0.922	< 0.001

**Fig. 6:**
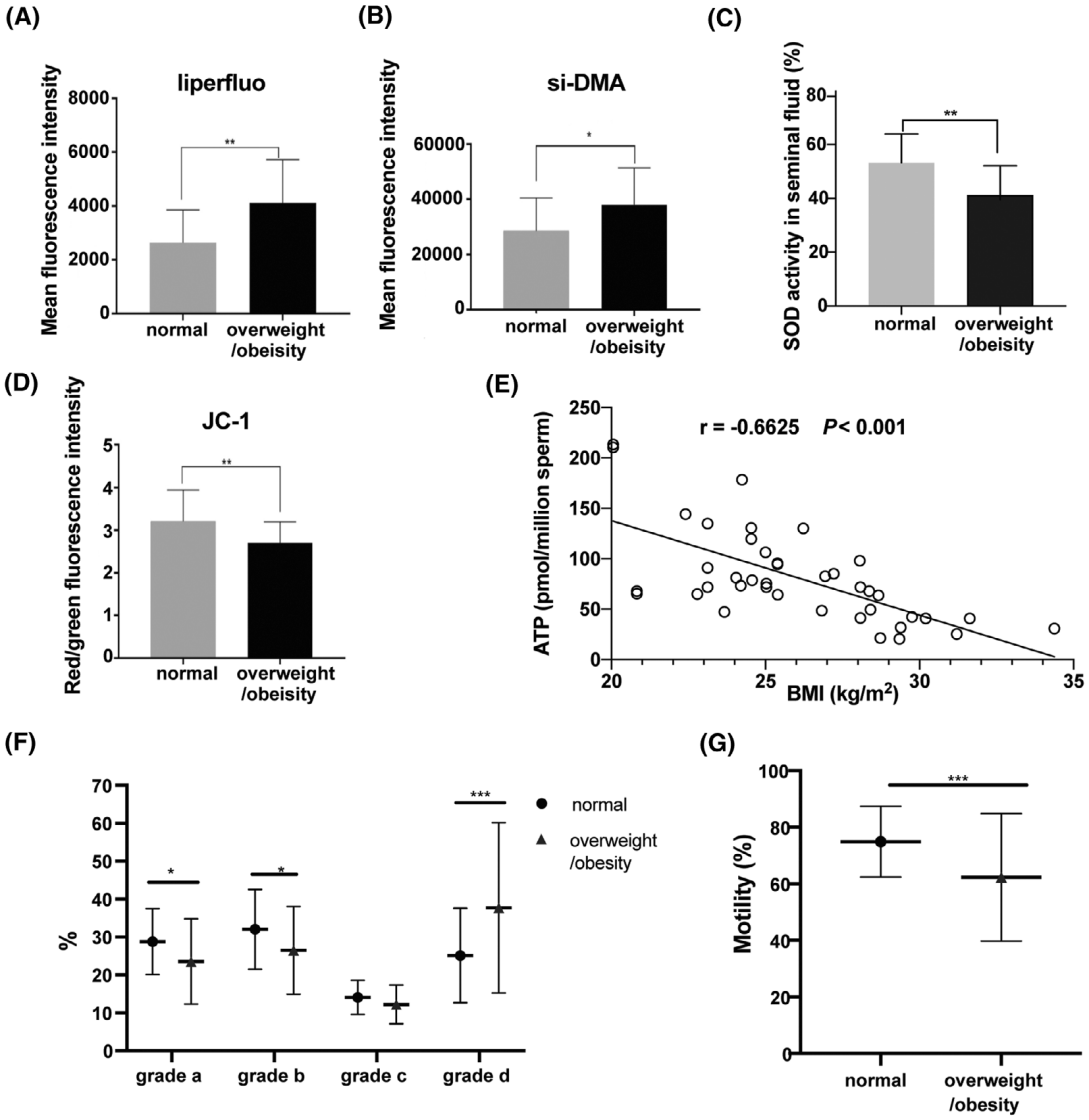
Oxidative stress and mitochondrial function measurements in human sperm. (A) Mean fluorescence intensity of Liperfluo (lipid peroxidation) and (B) si‐DMA (mitochondrial singlet oxygen) of human sperm from normal weight and overweight/obese groups (*n* = 15). (C) SOD activity was measured in human seminal plasma (*n* = 15). (D) Comparisons of the JC‐1 red/green fluorescence ratio in sperm between the two groups indicated mitochondrial membrane potential (*n* = 15). (E) Negative correlation between ATP content in human sperm and BMI values (*n* = 40). (F) Analysis of sperm quality via detecting the percentages of grade a, b, c, and d sperm, and (G) comparison of sperm motility between normal weight and overweight/obese subjects (*n* = 46 in normal weight group, *n* = 69 in overweight/obese group). Data are expressed as mean ± SD or percentage (%). Statistical analyses were performed using the Student's *t*‐test (A–D, F, and G) and Pearson correlation coefficient (E). *P < 0.05, **P < 0.01, ***P < 0.001. Grade a: linear motion; Grade b: slow motion; Grade c: *in‐situ* motion; Grade d: no motion. Motility (%) = grade a (%) + grade b (%).

We evaluated directly whether or not rises in the BMI levels in the overweight/obese group with rises in oxidative stress. Such an assessment stemmed from the realization that the HFD in mice showed increased lipid peroxidation, disrupted mitochondrial function, and reduced sperm quality. More direct evidence of an increase in oxidative stress was realized through measurements of lipid peroxidation and mitochondrial singlet oxygen generation and mitochondrial membrane potential of sperm along with superoxide dismutase activity (SOD) levels in the seminal fluid. Lipid peroxidation and mitochondrial singlet oxygen in sperm significantly increased, respectively (Fig. 6A,B), and the activity of SOD in seminal plasma decreased in overweight/obese men compared with that in normal weight men (Fig. 6C). JC‐1 staining also indicated a lower mitochondrial membrane potential in sperm from overweight/obese men (Fig. 6D). Moreover, there was a negative correlation between sperm ATP content and BMI with corresponding subjects (*r* = −0.66, *P* < 0.001, Fig. 6E). Sperm motility significantly decreased in overweight/obese men compared with that in normal weight men (62.29 ± 22.46 vs. 74.88 ± 12.48%, *P* < 0.001, Fig. 6G). Such declines were accompanied by decreases in sperm quality since the percentage of sperm assigned to the grade a and grade b groups decreased and were instead placed in the immotile d groups. Their rankings are classified here: grade a: 23.55 ± 11.22% vs. 28.77 ± 8.7%, *P* < 0.01; grade b: 26.51 ± 11.66% vs. 32.04 ± 10.54%, *P* < 0.05, and increased percentage of grade d sperm (37.71 ± 22.46% vs. 25.12 ± 12.46%, *P* < 0.001; Fig. 6F). Taken together, these results strongly suggest that increased oxidative stress and impaired mitochondrial function with declined ATP production in sperm from overweight/obese men were correlated with declined sperm motility, which is in agreement with the findings described in the HFD obese mouse model.

## Discussion

Overweight and obesity are defined as abnormal or excessive fat accumulation that may impair health [28]. In both human and animal experiments, obesity had negative effects on sperm quality and fertilization due to declines in sperm concentration, sperm motility, and total motile sperm count [29, 30, 31]. In the current study, semen samples from 69 overweight/obese Chinese men had significantly decreased sperm motility as indicated by a small percentage of grade a sperm, as well as an increased percentage of immotile grade d sperm in the overweight/obese group than in the normal group. Nevertheless, the sperm concentrations in the normal weight and overweight/obese groups were not different from one another. One possible reason for the sperm concentration to remain constant is that the number of individuals in the overweight/obese group whose BMI was excessively high was not large enough to significantly reduce the mean value of this group. It is conceivable that the sperm concentration only markedly declined in individuals with BMIs much larger than 30 kg·m^−2^.

To gain insight into the underlying mechanism that accounts for how obesity reduces sperm motility and quality, we established a high‐fat diet‐induced obese mouse model. In our case, there was a large enough difference between the caloric intakes in the two groups to cause weight gain to be driven by the higher fat content of the HFD. Such a difference was evident upon examination of the mesenteric adipose tissue, inguinal adipose tissue, retroperitoneal adipose tissue, and epididymal adipose tissue surrounding reproductive organs, which was in accordance with the HFD‐induced obese model in rats [16]. Moreover, higher levels of serum lipids and fatty liver were also observed in the HFD mice. However, the serum ALT levels were lower in the liver index of the HFD mice. This difference may result from placing them earlier and for a shorter term on the HFD that lasted from 4 to 14 weeks of age. Published data show that the extent of metabolic damage in response to HFD feeding is different between young and aged C57BL/6J mice. The older mice fed for 12 weeks an HFD starting at 44 weeks of age had significantly higher levels of serum ALT than those in the age‐matched control group, but the ALT levels were slightly decreased in the young mice fed an HFD from 6 weeks of age for 12 weeks than those in the age‐matched control group [32]. Besides, a large number of studies have shown that high levels of circulating lipids generally increase the availability of sources of metabolic energy for synthesis in anabolic metabolic pathways in adipocytes and nonfat cells, which in turn also increases ROS production. On the contrary, in this environment chronic inflammation can develop which underlies a host of systemic diseases that can promote oxidative stress and disrupt the generation of high‐quality sperm [33].

Oxidative stress is usually caused by increased ROS production and decreased antioxidant capacity as a consequence of exposure to exogenous or endogenous factors such as smoking, drinking, obesity, inflammation etc. [33]. Excessive ROS can oxidize lipids, protein, and DNA in germ cells and in turn contributes to low fertility rate or infertility [34]. In the current study, changes in MDA content, which is a by‐product of lipid peroxidation, serve as a biomarker of lipid peroxidation [35]. Its content significantly increased in serum from HFD mice, which reflects a rise in lipid peroxidation activity. On the contrary, the level of oxidative stress is reflective of a balance between oxidative and antioxidative enzymatic activities. Therefore, some antioxidant enzymes were detected in the mouse testis and epididymis. The results showed that the mRNA expression levels of GPX and CAT decreased in testes and caput epididymis in HFD mice, and both gene expression and protein levels of GPX decreased in cauda epididymis that contains mature sperm. Moreover, the activity of SOD was lower in testes and caput epididymis in HFD mice, which was in accordance with the results in seminal fluid from overweight/obese men compared with that in normal weight men. Thus, some healthcare providers encouraged male infertility patients to follow an antioxidant regimen for clinical treatment that included recommendations on how to establish a healthier lifestyle to reduce obesity‐induced oxidative stress damage and improve sperm quality [36, 37].

Even though MDA content is a relevant indicator of lipid peroxidation, its conventional measurement takes a long time and cannot be incubated with live cells for making rapid measurements [22]. Therefore, a suitable method was needed that can quickly, easily, and accurately detect the oxidative stress in mature sperm from the epididymis. Liperfluo is a new fluorescence probe for directly detecting lipid peroxidation in live cells, which can be used to detect early lipid peroxidation compounds [38]. Singlet oxygen (^1^O_2_) generation is one of the major mitochondrial ROS and can be detected by si‐DMA fluorescence probe [39]. In HFD mice, the rises detected by Liperfluo were consistent with those detected with the Si‐DMA probes and both were focused on the middle piece of the sperm, which is the location of mitochondrial sheath.

Mitochondria play roles in providing and regulating energy availability for enabling sperm flagella movement. In other words, sperm motility and subsequent fertilization are largely dependent on sperm mitochondrial function. The mitochondrial membrane potential (MMP) is generated by proton pumps (complexes I, III, and IV) during oxidative phosphorylation [24]. In the current study, the MMP of spermatozoa from both HFD mice and overweight/obese patients is significantly lower than that in the control group. This decline was accompanied by impairment of the mitochondrial structure including structural swelling and increased vacuolar content. Notably, damage to mitochondrial membrane will dissipate the MMP and decrease in turn ATP availability. Moreover, our proteomics analysis of differentially expressed proteins of sperm relevant to mitochondrial function revealed upregulation of proteins that are mainly involved in the oxidative phosphorylation status and may induce higher ROS levels in HFD‐fed mice. These effects may be a compensatory mechanism that offsets declines in energy transduction. Therefore, both HFD mice and overweight/obese men had a negative impact on mitochondrial structure and function, and finally decreased sperm quality and fertility.

AMPK can be activated by increases in AMP/ATP ratio to in turn promote increases in ATP production [26]. In addition, rises in mitochondrial ROS generation will stimulate an adaptive antioxidant response through the regulation of cellular metabolic balance [27]. This activation is generally triggered via phosphorylation of the Thr172 residue on AMPK (catalytic alpha subunit) [40]. In the present study, we found that the increase in the Thr 172 phosphorylation was significantly larger in the HFD mouse sperm than that in the CD mouse. It should be pointed out that the control of this phosphorylation response affects both AMPK activity and directional sperm movement. Several studies demonstrated that increased AMPK activation can induce increases in lipid disorders in sperm plasma membrane and in turn reduce sperm motility as well through phosphorylation of downstream protein substrates localized in sperm flagellar axoneme or other related structures for its motility [41, 42, 43]. These results are consistent with our data, which showed that poor sperm motility was evident in both the obese mouse model and overweight/obese subjects.

There are also some limitations in this study. The electron leakage during mitochondrial respiratory chain activity is one of the major sources of superoxide production [23]. Excessive ROS levels can attack the mitochondrial inner membrane [44], which in turn impairs mitochondrial function. These dysfunctional mitochondria produce even more ROS, resulting in a detrimental feedback loop [45]. Whether increased oxidative stress is upstream of mitochondrial impairment or simply a subsequent downstream response to preceding changes is still not clear. To address this question, mitochondria‐targeted antioxidants should be further used in HFD‐fed mice to see whether antioxidants can ameliorate declines in MMP and offset compromised sperm motility and finally to clarify the mechanisms accounting for how changes in ROS levels modulate sperm motility.

**Fig. 7:**
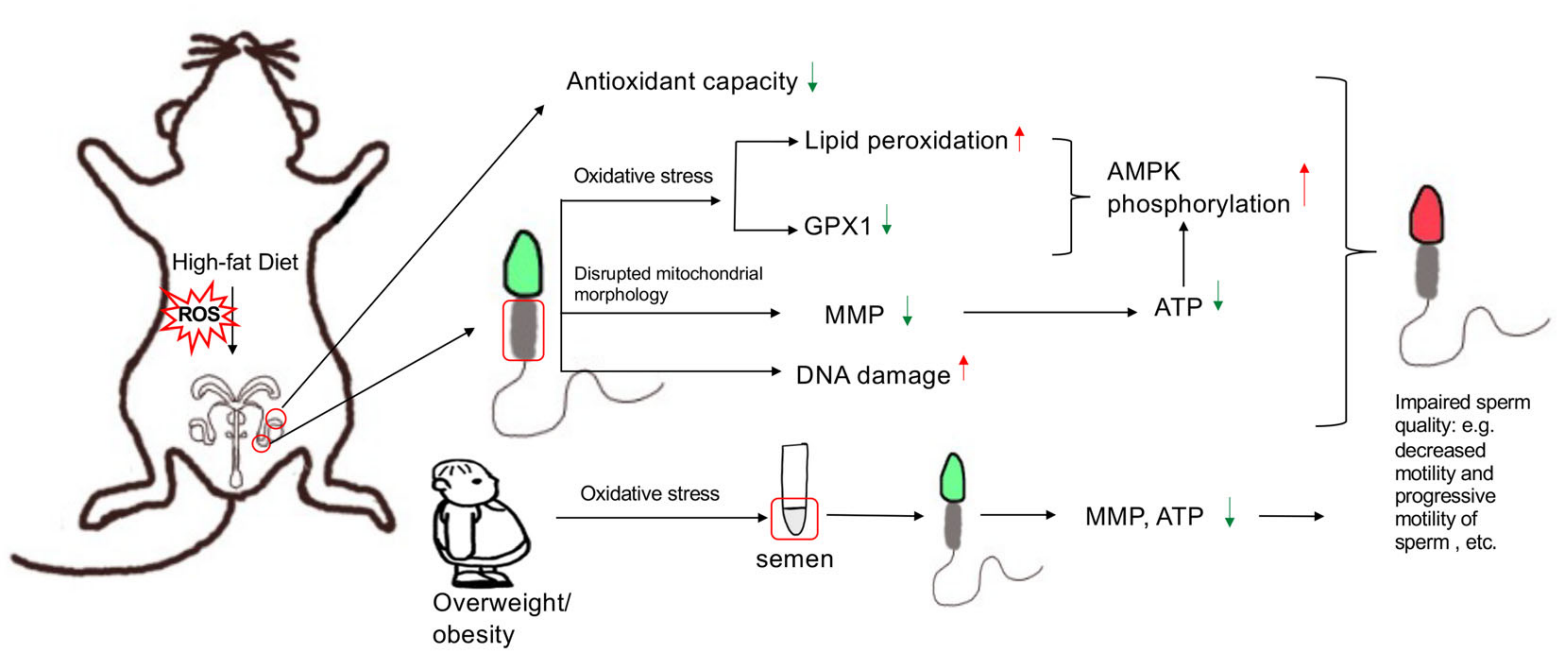
Schematic representation of impairment in structure and function of mouse and human sperm mitochondria by obesity‐induced oxidative stress.

In conclusion, we used a high‐fat diet‐induced obese mouse model to characterize the oxidative stress and impaired mitochondrial structure and function that underlie defective sperm quality. This semblance between these overweight/obese human subjects and the mice in the HFD group suggests that increases in oxidative stress may damage mitochondrial membrane and dissipate the mitochondrial membrane potential that is coupled to ATP generation. On the contrary, increased AMPK phosphorylation status at Thr172 also contributed to declines in sperm motility and ultimately male subfertility or infertility (Fig. 7). This research provides relevant insight showing that further studies are warranted to pinpoint targets controlling sperm oxidative stress status and MMP in overweight/obese individuals with subfertility in a clinical setting.

## Conflict of interest

The authors declare no conflict of interest.

## Author contributions

JJ and YP performed experiments and analyzed experimental data, and prepared the initial manuscript. WF collected the semen samples and analyzed the clinical data. SH, QP, CX, and XQ conducted part of molecular experiments. YL and ZD designed and supervised the project, and provided final approval of the manuscript. All authors read and agreed on the final version of the manuscript.

## Data Availability

All raw data supporting the conclusion of this research are available from the corresponding authors upon reasonable request.
